# Incidence and severity of myofiber branching with regeneration and aging

**DOI:** 10.1186/2044-5040-4-9

**Published:** 2014-05-15

**Authors:** Christophe Pichavant, Grace K Pavlath

**Affiliations:** 1 Department of Pharmacology, Rollins Research Center, Emory University, 1510 Clifton Road, Atlanta, GA 30322, USA

**Keywords:** Aging, *mdx*, Muscle injury, Muscle regeneration, Muscular dystrophy, Myofiber branching

## Abstract

**Background:**

Myofibers with an abnormal branching cytoarchitecture are commonly found in muscular dystrophy and in regenerated or aged nondystrophic muscles. Such branched myofibers from dystrophic mice are more susceptible to damage than unbranched myofibers *in vitro*, suggesting that muscles containing a high percentage of these myofibers are more prone to injury. Little is known about the regulation of myofiber branching.

**Methods:**

To gain insights into the formation and fate of branched myofibers, we performed in-depth analyses of single myofibers isolated from dystrophic and nondystrophic (myotoxin-injured or aged) mouse muscles. The proportion of branched myofibers, the number of branches per myofiber and the morphology of the branches were assessed.

**Results:**

Aged dystrophic mice exhibited the most severe myofiber branching as defined by the incidence of branched myofibers and the number of branches per myofiber, followed by myotoxin-injured, wild-type muscles and then aged wild-type muscles. In addition, the morphology of the branched myofibers differed among the various models. In response to either induced or ongoing muscle degeneration, branching was restricted to regenerated myofibers containing central nuclei. In myotoxin-injured muscles, the amount of branched myofibers remained stable over time.

**Conclusion:**

We suggest that myofiber branching is a consequence of myofiber remodeling during muscle regeneration. Our present study lays valuable groundwork for identifying the molecular pathways leading to myofiber branching in dystrophy, trauma and aging. Decreasing myofiber branching in dystrophic patients may improve muscle resistance to mechanical stress.

## Background

Muscular dystrophies are characterized by progressive rounds of muscle degeneration and regeneration (for review, see [1]). Muscle becomes aberrant over time, and various abnormalities, such as variations in myofiber size, decreased myofiber number, fibrosis and branched myofibers, are observed. Branched myofibers are malformed myofibers, which, instead of having a normal cylindrical shape, contain one or more offshoots of small daughter myotubes contiguous with the parent myofiber. Although branched myofibers have been reported extensively in the literature (for review, see [2]), little is known about the mechanisms involved in their formation or their long-term fate.

Despite the fact that branched myofibers are observed in several muscular dystrophies in human, dog, cat and mouse [3-11], myofiber branching has been studied predominantly studied in *mdx* mice, a mouse model of Duchenne muscular dystrophy (DMD) [10,12-15]. DMD is a genetic disease affecting about 1 in every 3,500 boys [16]. This X-linked disease is due to the absence of the sarcolemmal protein dystrophin in myofibers [17]. The lack of dystrophin leads to progressive muscle degeneration, and DMD patients usually die as a result of heart or respiratory failure during the third decade of life [16]. In *mdx* mice, Ca^2+^ signaling is altered in branched myofibers compared to unbranched myofibers, suggesting that contractile activity is impaired in branched myofibers [13]. Moreover, isolated branched myofibers of *mdx* mice are more susceptible to damage at the branch point in response to eccentric contraction [14,15,18], suggesting that muscles containing a high percentage of these myofibers are more prone to injury. Branched myofibers can also occur in nondystrophic muscles in response to aging, weightlifting or muscle injury induced by myotoxins [12,19-25], but only limited quantitative analyses of myofiber branching were performed in previously described experiments [12,19,24,25].

To gain critical insights into the formation and fate of branched myofibers, we performed in-depth quantitative analyses of myofiber branching in dystrophic and nondystrophic murine muscles. We compared the frequency of branched myofibers, the number of branches per myofiber and the morphologic features of branches in three different mouse models in which branching occurs. These models were *mdx* mice, and myotoxin-induced injury and aging in wild-type mice. We observed significant differences in myofiber branching among these three models. The incidence and severity of branching was highest in *mdx* muscles which undergo multiple rounds of degeneration and regeneration. Interestingly, commonly used myotoxins [26-28] such as barium chloride (BaCl_2_) or cardiotoxin (CTX) induced severe and irretrievable cytoarchitecture changes in regenerated myofibers of wild-type mice. Moreover, the morphology of the branched myofibers differed among the various models as well as over time. These studies lay valuable groundwork for gaining better understanding of the mechanisms underlying myofiber branching in dystrophy, trauma and aging.

## Methods

### Mice

Wild-type (C57BL/6) and *mdx* (C57BL/10) mice were purchased from The Jackson Laboratory (Bar Harbor, ME, USA). In all experiments, we used female and male mice at 8 to 12 weeks of age unless described otherwise. Experiments involving animals were performed in accordance with approved guidelines and ethical approval from Emory University’s Institutional Animal Care and Use Committee.

### Muscle injury

Mice were anesthetized with intraperitoneal injection of a solution containing 80 mg/kg ketamine HCl/5 mg/kg xylazine. For analgesia, mice were injected subcutaneously with 0.1 mg/kg buprenorphine before and after muscle injury. Injury was induced in the gastrocnemius (GA) muscles of anesthetized mice by injection of either 40 μl of 1.2% BaCl_2_[25] or 40 μl of 100 μg/ml CTX (Sigma-Aldrich, St Louis, MO, USA) [29]. Muscles were collected either 3 or 16 weeks after injury.

### Single-myofiber isolation

All muscles were uniformly processed for the three animal models studied: dystrophic, chemically injured and aged mice. Muscles were gently dissected and cut into three to five equal longitudinal pieces in order to increase the surface area of the muscle in contact with the enzyme and to liberate myofibers throughout the muscle. The pieces of muscle were put into a tube containing Dulbecco’s modified Eagle’s medium supplemented with 25 mM 4-(2-hydroxyethyl) piperazine-1-ethanesulfonic acid and 400 U/ml collagenase type I (Worthington Biochemical, Lakewood Township, NJ, USA), and then the tube was rocked at 26 rpm in an Enviro-Genie incubator (Scientific Industries, Bohemia, NY, USA) set at 37°C. Wild-type muscles were enzymatically digested for 80 to 90 minutes. Muscles from *mdx* and aged mice were digested for up to 120 minutes because these muscles were more difficult to digest, probably due to increased extracellular matrix (ECM), compared to wild-type muscles. After incubation in collagenase, single myofibers released from the digestion and the remaining pieces of muscles were washed three times to remove debris and then transferred to a clean Petri dish. Single myofibers were transferred into a 24-well plate coated with a gelatinous protein mixture (Matrigel; BD Pharmingen, San Diego, CA, USA) using a fire-polished Pasteur pipette. To increase the yield of myofibers, the remaining pieces of the digested muscle were triturated. One to two plates per muscle were filled with single, isolated myofibers yielding one to four myofibers per well within 1 hour of the enzyme treatment. Even with longer digestion, the number of single myofibers (mean ± SEM) isolated from GA muscles of *mdx* mice (55 ± 19 per animal, *n* = 23) or aged wild-type mice (79 ± 30 per animal, *n* = 6) was significantly less (*P* < 0.05) than the number isolated from chemically injured wild-type mice (118 ± 32 per animal, *n* = 16). Isolated myofibers were allowed to settle for 30 minutes in the well before the plates were centrifuged at 1,100 × *g* for 20 minutes and then fixed with 3.7% formaldehyde for 10 minutes.

### Single-myofiber analysis

Myofibers were stained with 1 μg/ml 4′,6-diamidino-2-phenylindole (DAPI; Sigma-Aldrich) for 2 to 3 minutes to visualize nuclei. Single myofibers were observed using an Axiovert 200 M microscope (Carl Zeiss Microscopy, Thornwood, NY, USA), and images were acquired using a 10× or 20× Plan-Neofluar lens objective (Carl Zeiss Microscopy) and camera (QImaging, Surrey, BC, Canada) with OpenLab 5.50 software (PerkinElmer, Waltham, MA, USA). All images were uniformly processed for size, brightness and contrast using Photoshop CS software (Adobe Systems, San Jose, CA, USA). Hypercontracted myofibers and myofibers less than 6 mm in length were not analyzed. Myofibers with at least four centrally located nuclei in a row were considered regenerated.

### Statistical analyses

To determine the statistical significance between two groups, comparisons were made using an unpaired Student’s *t*-test. χ^2^ tests were performed for the analysis of the number of branches per myofiber. The significance of the results obtained from multiple groups was evaluated by one-way analysis of variance with Bonferroni’s posttest correction. Statistical analyses were performed using GraphPad Prism v.4 software (GraphPad Software, La Jolla, CA, USA). A *P*-value <0.05 was considered significant.

## Results

### Analysis of myofiber branching in isolated myofibers

**Figure 1:**
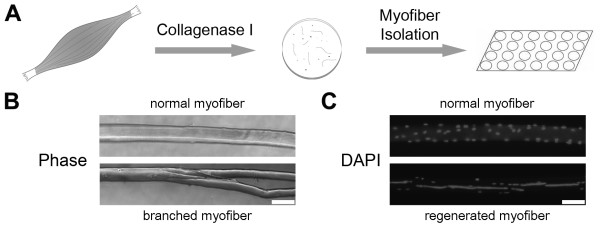
**Isolation of single myofibers. (A)** Muscles were digested with collagenase type I, and single myofibers were isolated. **(B)** A branched myofiber contains one or more small myotubes contiguous with the parent myofiber. Bar = 100 μm. **(C)** Myofibers were stained with 4′,6-diamidino-2-phenylindole (DAPI) to visualize nuclei. A myofiber with at least four centrally nuclei located in a row was considered regenerated. Bar = 100 μm.

To analyze and compare myofiber branching among dystrophic and nondystrophic mouse models, we studied isolated myofibers rather than transverse sections of muscle tissue. In transverse sections, myofibers must be individually tracked over long distances in numerous serial sections; thus, few myofibers can be analyzed, and branches cannot be accurately identified. In contrast, analysis of single, isolated myofibers is beneficial because large numbers of myofibers can be analyzed, and all branches along the length of the myofiber can be distinguished. In our studies, whole muscles were enzymatically digested, and single myofibers were isolated and fixed. Myofibers were then stained with DAPI to visualize nuclei and were examined using phase-contrast and fluorescence microscopy (Figure 1). To distinguish between regenerated and nonregenerated myofibers, myofibers with at least four centrally located nuclei in a row were scored as regenerated.

### Severity of myofiber branching increases with age in *mdx* mice

**Figure 2:**
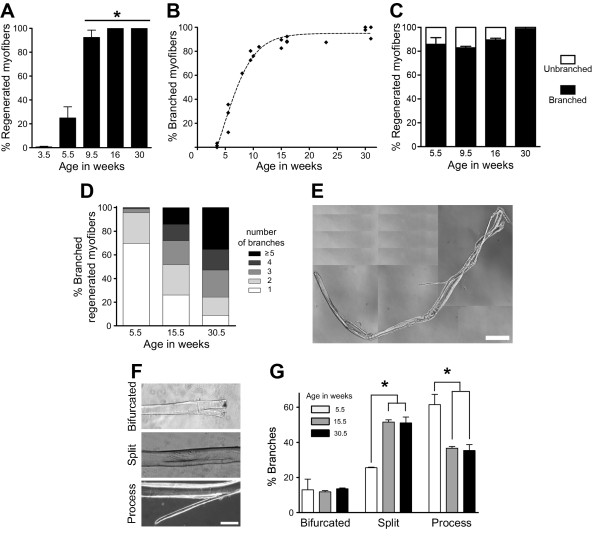
**Myofiber branching increases with age in *mdx *mice.** The percentage of regenerated myofibers **(A)** and branched myofibers **(B)** in gastrocnemius muscles from *mdx* mice rapidly increased between 3.5 and 9.5 weeks of age. **(C)** More than 80% of the regenerated myofibers were branched. **(D)** The number of branches per regenerated myofiber significantly increased with age in gastrocnemius muscles of *mdx* mice (χ^2^ = 82.7, *df* = 3). **(E)** Phase-contrast image of a branched myofiber from a 16-week-old *mdx* mouse. Bar = 250 μm. **(F)** Phase-contrast images of the three branching types studied. Bar = 100 μm. **(G)** Quantification of branching types in *mdx* mice at different ages. In **(A)**, **(C)**, **(D)** and **(G)**, 73 to 287 myofibers were analyzed per age, where 15.5 and 30.5 weeks are the averages of 15- and 16-week-old and 30- and 31-week-old mice, respectively. In **(B)**, each point represents one mouse with 16 to 103 myofibers. The dashed line represents the best fit curve for the percentage of branched myofibers with time. In **(A)**, **(C)** and **(G)**, data are mean ± SEM (*n* = 3 or 4 mice for all ages, except *n* = 2 for 9.5-week-old mice). *P < 0.05.

Muscles of *mdx* mice undergo repeated cycles of degeneration–regeneration between 3 and approximately 8 weeks of age [30,31]. To evaluate the short- and long-term effects of this extensive regenerative process on myofiber branching, we performed in-depth microscopic analyses of single myofibers isolated from GA muscles of 3.5- to 31-week-old *mdx* mice. The percentage of regenerated myofibers rapidly increased from about 1% to 90% between 3.5 and 9.5 weeks of age and then plateaued at 100% for mice older than 16 weeks (Figure 2A). During these same time frames, the percentage of branched myofibers also increased, reaching a plateau at approximately 90% in mice older than 16 weeks of age (Figure 2B). Notably, more than 80% of all regenerated myofibers were branched in *mdx* mouse GA muscles (Figure 2C), and 99% of all branched myofibers were regenerated (data not shown). The branches were always localized close to the regenerated region of the myofiber. These data indicate that the percentage of branched myofibers in muscles increases as a function of the proportion of regenerated myofibers.

To determine if myofiber branching becomes more severe with the number of degeneration–regeneration cycles in *mdx* mouse muscles, the number of branches was examined in regenerated myofibers. As shown in Figure 2D, the number of branches per regenerated myofiber significantly increased with age in *mdx* mice. The percentage of regenerated myofibers with one branch decreased from 69.7% to 8.8% over the 25-week period studied, whereas the percentage of regenerated myofibers with five or more branches increased from 0% to 35.4% during the same period of time. As a result, the average number of branches per regenerated myofiber increased from (mean ± SEM) 1.36 ± 0.04 at 5.5 weeks old to 4.04 ± 0.50 branches at 30.5 weeks old. As shown in Figure 2E, myofibers from older *mdx* mice predominantly displayed a very complex pattern of branching. Subsequently, the morphology of the branches in regenerated myofibers was analyzed as a function of age in *mdx* mice. Branches are usually grouped into three morphologic categories: bifurcated, split and process (Figure 2F), although the significance of these different types of branches is not understood [13,18]. The bifurcated and process types of branches are sprouts emanating from the myofiber. A bifurcated branch is localized at one of the extremities of the myofiber, whereas a process is not (Figure 2F, top and bottom). The split type of branch is a division or fissure of the myofiber into two segments and can be observed anywhere along the myofiber (Figure 2F, middle). Myofibers may have multiple branch types, as shown in Figure 2E. We observed no change in the percentage of bifurcated myofibers associated with age in GA muscles of *mdx* mice. However, the process morphology was most common at age 5.5 weeks, whereas split branches were most predominant in animals older than 15.5 weeks of age (Figure 2G). Together, these data (Table 1) indicate that the severity of myofiber branching, defined as the percentage of branched myofibers and the number of branches, increases with age and cycles of degeneration–regeneration in *mdx* muscles.

**Table 1:** **Summary data of myofiber branching in *mdx*, myotoxin-injured and aged wild-type mouse gastrocnemius muscles**

	**Young *mdx *** **(5.5 to 8 wk)**	**Adult *mdx *** **(30 to 31 wk)**	**Myotoxin-injured WT**^**a **^**(2 to 6 mo)**	**Aged WT**^**a **^**(20 to 21 mo)**
Regenerated myofibers/total myofibers^b^	38.6 ± 15.3	100 ± 0	69.2 ± 5.0	0
Branched myofibers/total myofibers^b^	34.7 ± 10.3	97.1 ± 2.3	38.2 ± 1.3	6.5 ± 0.6
Branched myofibers/regenerated myofibers^b^	85.8 ± 5.8	97.1 ± 2.3	55.4 ± 3.6	0
Number of branches per branched myofiber^c^	1.7 ± 0.3	4.0 ± 0.5	1.6 ± 0.1	1.0 ± 0

^a^WT, Wild type. ^b^Data are mean (%) ± SEM (*n* = 3 to 6 mice). ^c^Data are mean ± SEM (*n* = 3 to 6 mice).

### Chemical injuries induce myofiber branching in wild-type mice

The large increase in myofiber branching observed with aging in *mdx* mouse muscles may be influenced by the lack of dystrophin. To study myofiber branching purely as a function of muscle regeneration, we used chemical agents to induce muscle regeneration in wild-type mice. BaCl_2_ and CTX are two agents commonly used to induce muscle regeneration in rodents [26-28]. BaCl_2_ increases intracellular K^+^ uptake in myofibers, whereas CTX forms pores in the sarcolemma [32,33]. Although these two agents have disparate mechanisms of action, they both ultimately lead to necrosis of the myofiber by depolarization and degradation of the sarcolemma and activation of Ca^2+^-dependent proteases [34,35]. Myofiber branching induced by these two agents was subsequently analyzed and compared.

**Figure 3:**
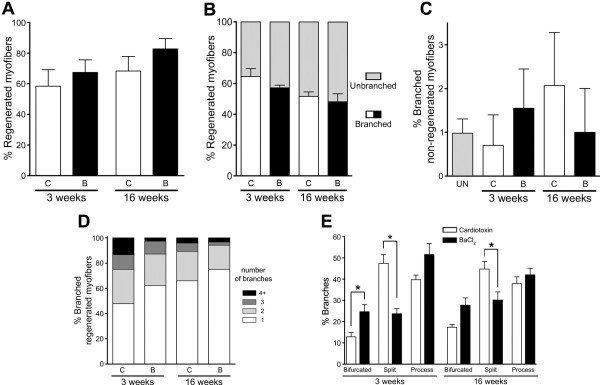
**Extensive myofiber branching occurs in regenerating wild-type muscles, regardless of the injury method used. (A)** No significant differences occurred in the percentage of regenerated myofibers at 3 or 16 weeks after injury induced by either cardiotoxin (C or CTX) or barium chloride (B or BaCl_2_) in adult wild-type gastrocnemius muscles. **(B)** The percentage of branched, regenerated myofibers was similar, regardless of the method used to induce injury, and did not change significantly with time after injury. **(C)** Injury did not significantly increase myofiber branching in nonregenerated myofibers. UN, Uninjured muscles. **(D)** The majority of branched, regenerated myofibers contained one branch. The number of branches per regenerated myofiber significantly differed between the two myotoxins at 3 weeks (χ^2^ = 10.4, *df* = 3), but not at 16 weeks, after injury (χ^2^ = 4.6, *df* = 3). The average number of branches per regenerated myofiber varied between 1.4 and 2 for all injuries and time points (CTX at 3 weeks = 2.0 ± 0.2, CTX at 16 weeks = 1.5 ± 0.1, BaCl_2_ at 3 weeks = 1.5 ± 0.1 and BaCl_2_ at 16 weeks = 1.4 ± 0.1). **(E)** Quantification of branching types at 3 and 16 weeks after injury (*n* = 252 to 645 myofibers isolated per injury method and time point). Data are mean ± SEM, with three to six injured mice and nine uninjured mice. *P < 0.05.

GA muscles of adult wild-type mice were injured by a single injection of BaCl_2_ or CTX, and, 3 weeks after injury, individual myofibers were isolated, fixed, stained and analyzed as described above for *mdx* myofibers. At 3 weeks postinjury, we observed no significant difference between the two myotoxins regarding the percentage of regenerated myofibers (Figure 3A), indicating that they caused a similar amount of muscle damage. Remarkably, approximately 60% of the regenerated myofibers were branched with either agent (Figure 3B). In contrast, only about 1% to 2% of nonregenerated myofibers from injured muscles were branched, which is similar to our observations in uninjured muscles (Figure 3C). These data indicate that branching is greatly induced in response to muscle regeneration and is restricted to regenerated myofibers. To normalize for small differences in the number of regenerated myofibers, we focused our further analyses of myofiber branching on regenerated myofibers. To evaluate the severity of the branching, the number of branches per myofiber was enumerated. Even though the majority of the regenerated myofibers contained one branch for both chemical agents (Figure 3D), the number of branches per myofiber was significantly different between CTX and BaCl_2_ at 3 weeks postinjury. Compared to BaCl_2_-injured muscles, a decrease in myofibers with one branch and an increase of myofibers with four or more branches were seen in CTX-injured muscles. The morphology of branched regenerated myofibers was also analyzed as described above for *mdx* mouse myofibers (Figure 2G). The percentage of bifurcated branches was significantly higher in BaCl_2_-injured muscles than in CTX-injured muscles, but the percentage of split branches was lower (Figure 3E). In contrast, the percentage of process branches was not significantly different.

To establish the fate of the branched regenerated myofibers at late times after injury, a single injection of BaCl_2_ or CTX was performed in GA muscles and then individual myofibers were isolated 16 weeks postinjury and analyzed as at 3 weeks postinjury. Except for the increase in split branches in CTX-injured muscles already observed at 3 weeks postinjury, no significant differences in myofiber branching were observed between BaCl_2_- and CTX-injured muscles at 16 weeks postinjury (Figures 3A to 3E). Subsequently, we compared the various parameters of branching between 3 and 16 weeks postinjury. A significant change noted was a modest decrease in the number of branches in BaCl_2_-injured muscles with time. These data suggest that the overall proportion of branched myofibers is stable with time in wild-type muscles, even though the number of branches can decrease under some injury paradigms. Together, these data (see Table 1) indicate that myotoxins commonly used to induce and study muscle regeneration in rodents lead to large amounts of branched regenerated myofibers.

### Myofiber branching increases with age in wild-type mice

**Figure 4:**
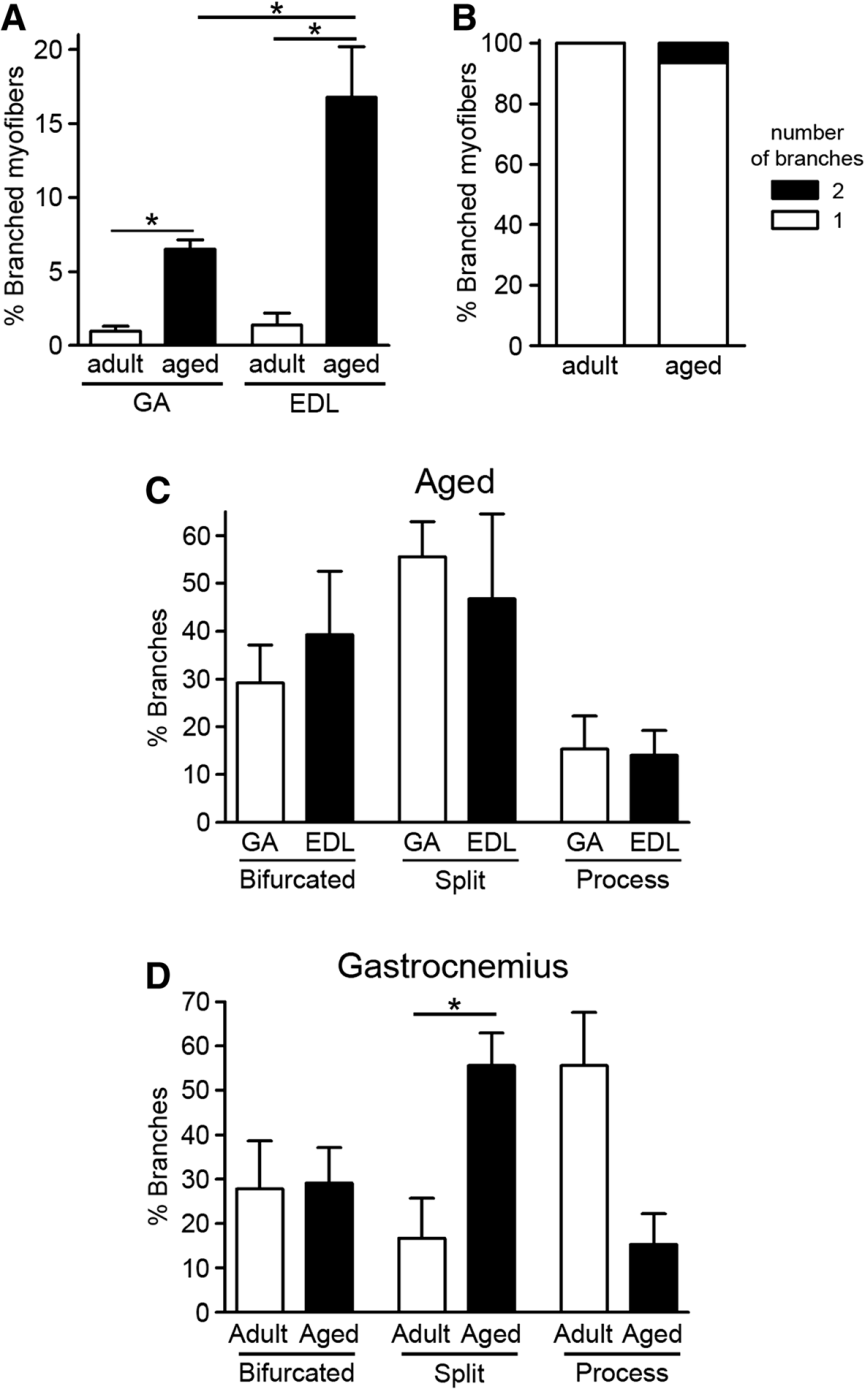
**Myofiber branching increases with normal aging in wild-type mice. (A)** In both gastrocnemius (GA) and extensor digitorum longus (EDL) muscles, myofiber branching increased with age in wild-type mice. **(B)** The majority of the branched myofibers from aged muscles contained only one branch. Data for GA and EDL muscles are pooled. **(C)** No significant differences were observed in branch types between GA and EDL muscles of aged mice. **(D)** The percentage of split branches increased with age in GA muscles. In all graphs, the adult mice were 2 to 6 months, and aged mice were 20 to 21 months old. In **(A)** and **(C)**, 96 to 596 myofibers were analyzed per age and data are mean ± SEM of 4 to 31 mice. *P < 0.05. Data in **(B)** represent 21 to 46 branched myofibers from pooled GA and EDL muscles per age expressed as means of 10 to 35 mice. Data in **(D)** represent 18 to 31 branched myofibers from GA muscles expressed as mean ± SEM of 6 to 18 mice.

Muscle mass and function are decreased with aging, causing weakness [36,37]. To determine if aging also influences myofiber branching, we investigated branching in adult and aged wild-type mice. Myofiber branching in GA and extensor digitorum longus (EDL) muscles from adult (2- to 6-month-old) and aged (20- to 21-month-old) wild-type mice were analyzed as described for *mdx* and injured wild-type muscles. In both muscles, the percentage of branched myofibers significantly increased by six- to twelvefold with age, with a greater increase observed in EDL muscles (Figure 4A). After DAPI staining of myonuclei, none of the isolated aged myofibers contained centrally located nuclei (data not shown). The number of branches per branched myofiber was also calculated to determine the severity of the branching. The majority of the branched myofibers in aged GA and EDL muscles contained only one branch (Figure 4B). The morphology of the branched myofibers was also analyzed as described above for *mdx* and injured wild-type muscles. As shown in Figure 4C, aged GA and EDL muscles displayed similar percentages of the three types of branches. The incidence of the split branch type was significantly increased in aged GA myofibers in comparison to adult GA (Figure 4D). A comparison of branch types between adult and aged EDL was not possible because of the low number of branched myofibers isolated from this muscle in adult mice. These data (Table 1) demonstrate that aging induces the formation of branched myofibers in wild-type muscles, though its incidence and severity are much lower than in adult *mdx* muscles or myotoxin-injured wild-type muscles.

## Discussion

**Figure 5:**
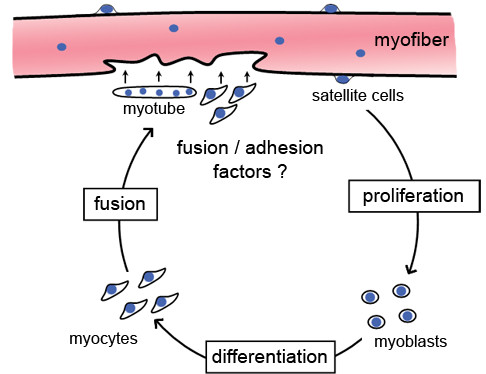
**Illustration of the myofiber branching model.** After muscle injury, satellite cells proliferate, differentiate and fuse either directly to the damaged myofiber or with each other to form myotubes. Subsequently, these myotubes may also fuse with the damaged area to repair the myofiber. A defect in adhesion or fusion during muscle regeneration may lead to branched myofibers.

Little is known about how branched myofibers arise in different pathologic and physiologic conditions such as neuromuscular disease, weightlifting or muscle trauma [4,38]. Two hypotheses have been put forth to explain the origin of these abnormal myofibers. The first one postulates that branched myofibers result from myofibers’ undergoing longitudinal tearing. This theory is used mostly to explain myofiber branching due to hypertrophy [22,39]. The second hypothesis proposes that myofiber branching arises from the imperfect fusion of myogenic cells during muscle regeneration (Figure 5) [13,18,40]. Indeed, during regeneration, the adhesion and/or fusion of either satellite cells or small myotubes formed within the old basal lamina surrounding injured myofibers could be altered [20,40-42]. As discussed below, our studies of myofiber branching in dystrophic and nondystrophic muscles support the hypothesis that myofiber branching arises during muscle regeneration.

Myofiber branching was previously observed and quantified on myofibers isolated from *mdx* mice [12,13,15]. The maximum amount of branching observed differed, depending on the muscle analyzed. In *mdx* mouse EDL muscles, branching increased with age, such that 65% to 100% of myofibers were ultimately branched [12,13,15], whereas only 11% of flexor digitorum brevis myofibers became branched [13]. Differences were noted at the age, between 4 and 38 weeks old, when the maximum amount of branching was observed. Although these studies demonstrated an increase in myofiber branching over time in *mdx* mice, they did not analyze the average number of branches with age, nor did they seek to determine whether myofiber branching occurred in regenerated myofibers. In contrast to these studies, we performed in-depth analyses of the frequency of branching in regenerated myofibers, the number of branches per myofiber and the morphologic features of branches in young and adult *mdx* mice to better understand the mechanisms underlying the generation of branched myofibers. We found that the percentage of branched myofibers increased as a function of the proportion of regenerated myofibers, such that, by 30 weeks of age, 99% of branched myofibers were regenerated in GA muscles. Even though *mdx* mouse muscles undergo fewer cycles of degeneration–regeneration after about 8 weeks of age [30,31], the number of branches per branched myofiber still increased with age. These data indicate that myofiber branching is an ongoing process occurring in *mdx* mouse muscles. Intriguingly, the morphology of the branches changed with age in *mdx* mouse muscles. The percentage of the split type of branch increased between 5.5 and 15.5 weeks of age, whereas the percentage of the process type of branch decreased. In contrast, the percentage of bifurcated branches did not change during this 10-week period. Because degeneration–regeneration cycles in *mdx* mouse muscles are not synchronized and myofiber regeneration in mice is mostly complete in approximately 2 to 3 weeks, not all of the branched, regenerated myofibers analyzed in 5.5-week-old *mdx* mice were likely fully regenerated. Thus, the type of branches observed in isolated myofibers from 5.5-week-old *mdx* mice may be temporary if developing branches fuse back completely or partially with the main myofiber at later times.

Myofiber branching may be exacerbated in *mdx* mice by the increase in ECM that occurs with age [43] or by the absence of dystrophin, which leads to mislocalization or absence of some members of the dystrophin–glycoprotein complex, such as nitric oxide synthase [44,45]. In order to examine myofiber branching in nonpathologic muscle degeneration–regeneration cycles, we also performed in-depth analyses of myofiber branching in myotoxin-injured muscles of adult wild-type mice. Wild-type muscles were injured with either BaCl_2_ or CTX, two myotoxins commonly used to study muscle regeneration in mice. Three weeks after muscle injury, approximately 98% to 99% of all branched myofibers were regenerated. These data support the hypothesis that branched myofibers form as a result of myofiber regeneration. Interestingly, the same low amount of branching (1% to 2%) was found in nonregenerated myofibers isolated from either uninjured or injured muscles, suggesting that the extracellular milieu present during muscle degeneration–regeneration does not induce branching in uninjured myofibers. This low level of branching in nonregenerated myofibers corresponds to the basal level of branching believed to occur during muscle development [21,46,47]. Not all regenerated myofibers were branched following muscle injury. Formation of branches is likely proportional to the severity of injury to a particular myofiber. A small injury could be sufficient to result in four centrally nucleated nuclei (our definition of a regenerated myofiber), but not enough to induce the formation of a branched myofiber. The cytoarchitecture of regenerated muscles is typically believed to be similar to that of uninjured muscles [26-28]. However, we found that 55.4% of regenerated myofibers were branched even 4 months after injury, suggesting that mature branches, once formed, do not fuse with parent myofibers. This discrepancy may be due to the fact that muscle regeneration is studied mainly on muscle sections; however, by analyzing isolated myofibers, we were able to visualize small cytoarchitectural changes that are more difficult to observe on muscle sections. In addition, the fact that more than half of regenerated myofibers are branched may explain why researchers in some previous studies who analyzed tissue sections reported myofiber hyperplasia after myotoxin injury [48-50], as branches would appear to be additional myofibers. For both BaCl_2_ and CTX injuries, the overall incidence of branched, regenerated myofibers was comparable at 3 and 16 weeks postinjury and did not change with time. However, a few differences were noted between these two myotoxins. For example, the number of branches per myofiber significantly differed at 3 weeks, but not at 16 weeks, after injury. In addition, the split morphology predominated in CTX-injured muscles. These data suggest that both myotoxins induce a similar amount of damage to myofibers, but that the repair process may differ. Indeed, these myotoxins cause myofiber degeneration by disparate mechanisms of action [32-35] and may also differ in their impairment of satellite cells, the ECM or other components of the niche, with subsequent effects on myofiber branching.

We also investigated myofiber branching in a physiologic condition—aging—in wild-type mouse muscles. We observed an increase in the percentage of branched myofibers in aged GA and EDL muscles, with a threefold greater incidence in EDL muscles. Even though GA and EDL muscles are both hindlimb muscles, the different amounts of myofiber branching found in these two muscles could be explained by their different sizes or different fiber-type composition as well as by their roles in locomotion [51]. In contrast to *mdx* and myotoxin-injured wild-type mouse muscles, none of the branched myofibers in aged muscles were regenerated, defined as at least four centrally located nuclei in a row. Either myofiber branching is due to another mechanism than muscle regeneration in aged muscles or the central position of myonuclei is not a good marker of muscle regeneration at this time point. Indeed, the stability of centrally located nuclei in myofibers has been studied only up to 6 months postinjury [52]. No difference in the morphology of the branches was observed between aged GA and EDL muscles. However, an increase in the split type of branch occurred in GA muscles with age, as also found in *mdx* muscles. Satellite cell number and functionality are commonly thought to decline with age or dystrophy [53,54]; therefore, defects in myogenic cells might contribute to the increase in split branches.

A strength of our study lies in the ability to compare various measures of myofiber branching using standardized conditions between the three models examined: *mdx*, myotoxin-injured wild-type and aged wild-type muscles. Aged *mdx* mice exhibit the most severe myofiber branching, as defined by the incidence of branched myofibers and the number of branches per myofiber, followed by myotoxin-injured wild-type muscles and then aged wild-type muscles. Even though these branching parameters in chemically injured wild-type mice were similar to those of 5.5- to 8-week-old *mdx* mice, repetitive cycles of degeneration–regeneration in *mdx* muscles eventually led to more severe myofiber branching than a single injection of myotoxin into wild-type muscles. The robust incidence of myofiber branching in response to dystrophin deficiency or induced injury suggests that the quantification of myofiber branching may serve as an additional way to monitor the success of regenerative therapies, alongside the commonly used measurement of myofiber cross-sectional area.

A key question raised by these studies is whether muscles containing a high proportion of branched myofibers display altered function or increased susceptibility to mechanical stress *in vivo*. Branched myofibers isolated from dystrophic mice are weaker than nonbranched myofibers and are more susceptible to injury at the branch point [14,15,18]. The high number of branched myofibers in older *mdx* mice has been proposed as a contributor to muscle weakness in this model [18], but it is hard to separate the effects due to loss of dystrophin from the number of branched myofibers in whole muscles. In a regeneration study using normal rats, significant changes were observed in muscle function following repetitive bupivacaine-induced injury, with these differences being attributed to the presence of branched myofibers [55]. Whether the low incidence (2.5%) of branched fibers observed in the study by Tamaki *et al.* did actually contribute to these contractile changes is unknown. The effect of myofiber branching on whole-muscle function and susceptibility to injury should be clarified in future experiments.

## Conclusions

The strong association of branching with regenerated myofibers suggests that incomplete adhesion and/or fusion of myogenic cells occurs when large portions of a myofiber degenerate (Figure 5). This correlation is in agreement with the results of previous studies in which researchers analyzed myofiber branching in the context of muscle regeneration or myoblast transplantation [18,20,24,25,40,42,56]. Modulating the expression of molecules that regulate adhesion or fusion of myogenic cells *in vivo* (for review, see [57,58]) will provide further insights into the mechanisms by which branches form. A better understanding of the molecular pathways leading to myofiber branching may be beneficial for treating dystrophic patients by the development of therapeutic modalities to decrease myofiber branching and thus likely improve muscular resistance to mechanical stress.

## Abbreviations

CTX: Cardiotoxin; DAPI: 4′,6-diamidino-2-phenylindole; DMD: Duchenne muscular dystrophy; EDL: Extensor digitorum longus; GA: Gastrocnemius.

## Competing interests

The authors declare that they have no competing interests.

## Authors’ contributions

CP and GKP conceived of and designed the study. CP performed the research. CP and GKP analyzed the research and wrote the manuscript. Both authors read and approved the final manuscript.
